# Trends in Management of Ménière Disease: A TriNetX Network Database Analysis

**DOI:** 10.1002/oto2.123

**Published:** 2024-03-14

**Authors:** Tyler J. Gallagher, Meredith E. Adams, Janet S. Choi

**Affiliations:** ^1^ Keck School of Medicine of the University of Southern California Los Angeles California USA; ^2^ Department of Otolaryngology–Head and Neck Surgery University of Minnesota Minneapolis Minnesota USA; ^3^ Caruso Department of Otolaryngology–Head and Neck Surgery University of Southern California Los Angeles California USA

**Keywords:** endolymphatic sac surgery, guidelines, intratympanic injection, labyrinthectomy, ménière disease, treatment

## Abstract

This study investigated management practices for Meniere's disease (MD) and their temporal trends from 2008 to 2022 within the TriNetX network database. Study cohort included adult patients (≥18 years) with the diagnosis of MD from TriNetX's multi‐institutional medical records (n = 77,493). MD diagnosis and management were queried based on the international classification of diseases, tenth revision, current procedural terminology, and RXNorm codes. Temporal trends were analyzed using joinpoint regression. There was significant increase in rates of relevant medications prescribed within 12 months of MD diagnosis from 2008 to 2022 (annual percent change [APC]: 1.2 [95% confidence interval, CI: 0.4–1.9]). There were no significant changes in rate of intratympanic injection within 12 months of MD diagnosis (1.7 [95% CI: −1.1 to 4.5]). Rate of endolymphatic sac surgery and labyrinthectomy any time after MD diagnosis gradually decreased from 2008 to 2022 at APC of −8.1 (95% CI: −11.8 to −4.2) and −11.0 (95% CI: −14.0 to −7.7), respectively. Use of relevant medications has significantly increased during the early management of MD and the overall use of surgical treatments has decreased.

Management approaches for Meniere's disease (MD) have changed substantially over time. The American Academy of Otolaryngology updated clinical practice guidelines for MD in 2020 to improve diagnostic workup and treatment outcomes.^1^ While the guidelines provided an up‐to‐date summary of existing evidence, management strategies for MD remain complex with significant heterogeneity. Prior studies exploring MD management practices were based on data obtained prior to new guidelines and short‐term cross‐sectional data.^2^, ^3^, ^4^ Here, we investigated trends in management practices for MD, including the use of medications, intratympanic injection, and surgery, from 2008 to 2022.

## Methods

This is a multi‐institution retrospective chart review utilizing TriNetX, a globally federated health research network providing access to deidentified electronic medical records.^5^ Data in the Research network included >108 million patients across 76 Health Care Organizations (HCOs) and 4 countries (non‐US nations unidentifiable).^5^ Our cohort is primarily composed of patients from US tertiary care centers (96%). This study received exempt approval from the University of Southern California Institutional Review Board (UP‐23‐00652).

MD diagnosis, relevant medications, and procedures were assigned based on international classification of diseases, tenth revision, current procedural terminology, and RXNorm codes (Supplement Data SA, available online). Study cohort included adults (≥18 years) with MD diagnoses made between 2008 and 2022. Rates of relevant medications, intratympanic injection, endolymphatic sac surgery, and labyrinthectomy use within 12 months of diagnosis and any time after diagnosis were examined per 10,000 patients who received MD diagnoses each year. A 12‐month time point was applied for temporal trends analysis for medications and intratympanic injection a priori to understand the utilization of these management options during early periods.^6^, ^7^

SPSS (v28.0; IBM Corp) was used for descriptive statistics. Joinpoint Regression Program (v5.0.2; National Cancer Institute)^8^ was utilized to analyze annual percent change (APC) in treatments and identify significant changes in trends of treatment rates (“joinpoints”). Logarithms of the rates were utilized, with errors following a Poisson distribution. Settings allowed identification of 0‐to‐1 joinpoints. Significance was set at *α* = .05.

## Results

Cohort included 77,493 adults with MD diagnosis from 2008 to 2022 (age, mean [SD]: 65 [15]; Table 1). Rate of medication use per 10,000 within 12 months of MD diagnosis revealed highest use of diuretics, followed by benzodiazepines, antivertigo agents, and antihistamines (Table 2). Rate of intratympanic injection per 10,000 within 12 months of diagnosis was 364.5. Rates of endolymphatic sac surgery and labyrinthectomy per 10,000 any time after diagnosis were 87.6 and 38.5, respectively.

**Table 1:** Study Cohort Characteristics (n = 77,493)

Characteristics	N, percent of Ménière disease cohort (total N = 77,493)
Age,^a^ mean (SD)	65 (15)
Sex	
Male	22,778 (29.4%)
Female	49,679 (64.1%)
Unknown sex	5036 (6.5%)
Ethnicity	
Not Hispanic or Latino	52,299 (67.5%)
Unknown ethnicity	21,730 (28.0%)
Hispanic or Latino	3464 (4.5%)
Race	
White	59,562 (76.9%)
Unknown race	11,178 (14.4%)
Black or African American	3559 (4.6%)
Asian	2876 (3.7%)
American Indian or Alaskan Native	188 (0.2%)
Native Hawaiian or Other Pacific Islander	130 (0.2%)

Abbreviation: SD, standard deviation.

^a^ Current age, not age at diagnosis.

**Table 2:** Utilization Rate of Meniere's Disease Management Options Between 2008 and 2022 (Per 10,000 Patients Diagnosed With Meniere's Disease Within 12 Months and Per 10,000 Patients Diagnosed With Meniere's Disease at Any Time‐Point)

	Within 12 months of MD diagnosis (per 10,000 patients)	Any time since MD diagnosis (per 10,000 patients)
Oral medications^a^	5392.0	6812.0
Antihistamine	953.2	1872.1
Antivertigo Agent	1888.0	2761.5
Benzodiazapine	2587.3	4124.7
Diuretics	2958.6	3851.5
Intratympanic injection (corticosteroid or gentamicin)	364.5	484.3
Endolymphatic sac surgery	63.1	87.6
Labyrinthectomy	27.5	38.5

Abbreviation: MD, Meniere's disease.

^a^ Any medications defined as receiving one or more medications from the following groups—Antihistamines: Betahistine, Diphenydramine, Diphenhydrinate; Antivertigo Agents: Meclizine, Scopalomine; Benzodiazapines: Clonazepam, Diazepam, Lorazepam, Midazolam; Diuretics: Acetazolamide, Hydrochlorothiazide, Spironolactone, Triamterene.

**Figure 1:**
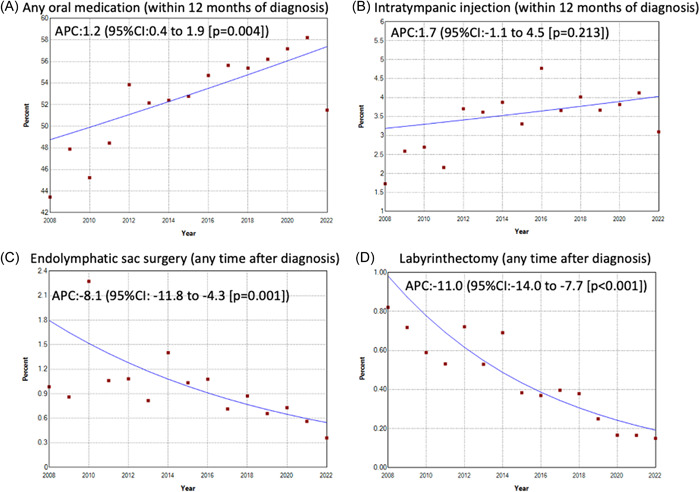
Joinpoint graphs demonstrating changes in rates (annual percent change) of treatments for Meniere's disease from 2008 to 2022. (A) Any oral medication (within 12 months of diagnosis), (B) Intratympanic injection (within 12 months of diagnosis), (C) Endolymphatic sac surgery (any time after diagnosis), and (D) Labyrinthectomy (any time after diagnosis). Rates defined by number diagnosed and receiving specified treatment divided by number diagnosed per year. APC, annual percent change; CI, confidence interval.

Joinpoint analysis demonstrated significant, positive APC for relevant medication use within 12 months of MD diagnosis over the period 2008 to 2022 (APC = 1.2, 95% confidence interval [CI]: 0.4–1.9). APC for intratympanic injection within 12 months of diagnosis was positive but the changes were not statistically significant (APC = 1.7, 95% CI: −1.1 to 4.5). APC for endolymphatic sac surgery and labyrinthectomy any time after MD diagnosis showed a significant, negative APC at −8.1 (95% CI: −11.8 to −4.3) and −11.0 (95% CI: −14.0 to −7.7), respectively without significant joinpoints (Figure 1). When a 2010 outlier in endolymphatic sac surgery was excluded, endolymphatic sac surgery was stable from 2008 to 2014 but decreased from 2014 to 2022 (Supplement Data SB, available online).

## Discussion

This analysis elucidated several trends in MD management. Medication use for MD steadily increased and utilization of intratympanic injection of steroids or gentamicin remained stable. Contrarily, the use of surgical interventions, including endolymphatic sac surgery and labyrinthectomy, decreased from 2008 to 2022.

As in prior studies, our findings demonstrated that oral medications for both symptom control and maintenance are commonly used during early periods after MD diagnosis^4^ and use has increased in the past 15 years. The 2020 AAO‐HNS clinical guidelines endorsed intratympanic steroid injection as optional and gentamicin injection as recommended management for MD patients with uncontrolled symptoms.^1^ While prior survey data reported increase in use of intratympanic gentamicin over the 1990s,^2^ we observed no change in intratympanic injection use from 2008 to 2022. This plateau in intratympanic injection usage is a novel finding and may represent general acceptance of the strategy in tertiary centers comprising the cohort. Academic practices were previously observed to utilize such procedural treatment more frequently for MD than community centers.^4^, ^9^ Analysis of treatment trends in community practices after guideline publication might reveal trends not observed here.

In contrast, we find that the use of surgical interventions has steadily decreased over time. AAO‐HNS guidelines emphasize surgical labyrinthectomy to be offered to a small subset of patients with persistent, symptomatic MD refractory to conservative treatments with nonusable hearing.^1^ No recommendation was made regarding the use of endolymphatic sac surgery due to its uncertain benefit and discordant study results. Our results suggest the decline of surgical management in recent years, likely reflecting the rise of less invasive treatment options and more aggressive medical management of acute symptoms.^3^, ^4^

Our study has limitations. Details regarding cohort composition and changes over the study period were uncertain due to blinding for anonymity. Individuals may have received treatment before presenting to an included HCO. Additionally, the cohort includes data primarily from tertiary centers in the United States and practices may differ in other settings. MD is a clinical diagnosis, which may result in misdiagnoses within this cohort. MD diagnosis and treatment codes were assigned by clinicians and inaccuracies in coding are possible Additionally, treatments were unable to be linked to MD diagnosis as the reason for treatment. Exact timing of treatments was unable to be determined.

## Conclusion

In this cohort, the use of medical management has significantly increased for the early management of MD, and the overall use of surgical treatments has decreased. Our findings imply an increasing use of less invasive approaches for MD management, in line with updated clinical guidelines.

## Author Contributions

**Tyler J. Gallagher**, conception and design of work, data acquisition, data analysis, interpretation of data, drafting of manuscript, critical revision; **Meredith E. Adams**, data analysis, interpretation of data, critical revision; **Janet S. Choi**, conception and design of work, data analysis, interpretation of data, drafting of manuscript, critical revision.

## Disclosures

### Competing interests

None.

### Funding source

None.

## Supporting information

Supporting information.

Supporting information.
