# Supplementary material for: Trends in Management of Ménière Disease: A TriNetX Network Database Analysis
Source: OTO Open. 2024 Mar 14;8(1):e123. doi: 10.1002/oto2.123 (PMC10938781; doi:10.1002/oto2.123)
Supplement: Supplementary file 1 — Supporting information. [file OTO2-8-e123-s001.docx]

**Supplement A:** ICD-10*, CPT, and RXNorm Codes

| **Code Type** | **Code** | **Diagnosis/Treatment** | **Drug Class (if applicable)** |
| --- | --- | --- | --- |
| ICD-10 | H81.01-H81.03 & H81.09 | Ménière’s disease | - |
| CPT | 69805, 69806 | Endolymphatic Sac Surgery | - |
|  | 69905, 69910 | Labyrinthectomy | - |
|  | 69801 | Intratympanic Injection (Gentamicin and Corticosteroid) | - |
| RXNorm | 1511 | Betahistine | Antihistamines |
|  | 3444 | Dimenhydrinate | Antihistamines |
|  | 3498 | Diphenhydramine | Antihistamines |
|  | 6676 | Meclizine | Antivertigo Agent |
|  | 9601 | Scopolamine | Antivertigo Agent |
|  | 2598 | Clonazepam | Benzodiazepines |
|  | 3322 | Diazepam | Benzodiazepines |
|  | 6470 | Lorazepam | Benzodiazepines |
|  | 6960 | Midazolam | Benzodiazepines |
|  | 167 | Acetazolamide | Diuretic |
|  | 5487 | Hydrochlorothiazide | Diuretic |
|  | 9997 | Spironolactone | Diuretic |
|  | 10763 | Triamterene | Diuretic |

*TriNetX utilized a 9-to-10-CM mapping system to transform data provided in ICD-9 to ICD-10 codes.
