# Supplementary material for: Trends in Management of Ménière Disease: A TriNetX Network Database Analysis
Source: OTO Open. 2024 Mar 14;8(1):e123. doi: 10.1002/oto2.123 (PMC10938781; doi:10.1002/oto2.123)

**Supplement B**: Joinpoint graph demonstrating changes in rates (annual percent change) of

endolymphatic sac surgery for Meniere’s disease from 2008 to 2022 without the outlier data

point in 2010.


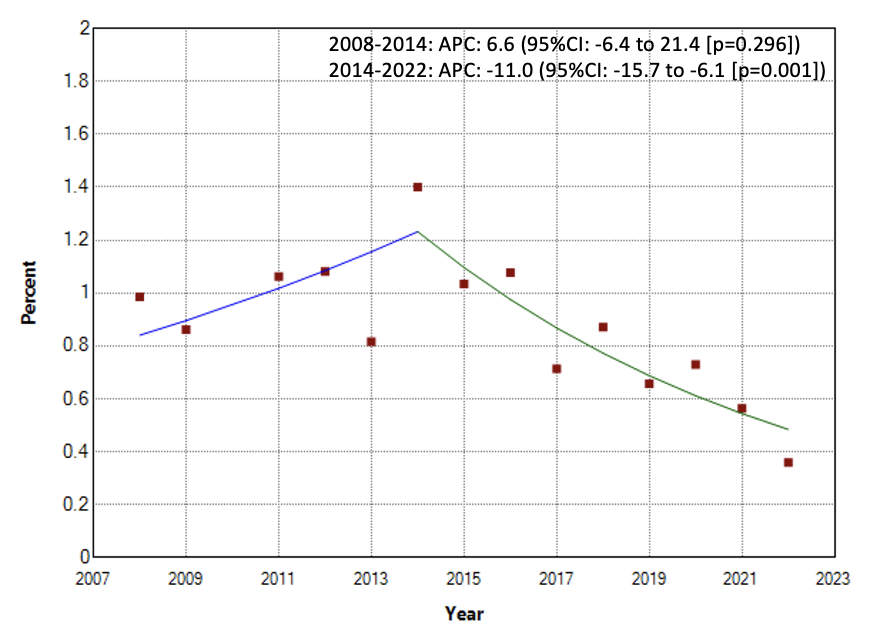

Supplement: Supplementary file 2 — Supporting information. [file OTO2-8-e123-s002.docx]
